# Phase 2 Randomised Controlled Trial and Feasibility Study of Future Care Planning in Patients with Advanced Heart Disease

**DOI:** 10.1038/srep24619

**Published:** 2016-04-19

**Authors:** Martin A. Denvir, Sarah Cudmore, Gill Highet, Shirley Robertson, Lisa Donald, Jacqueline Stephen, Kristin Haga, Karen Hogg, Christopher J. Weir, Scott A. Murray, Kirsty Boyd

**Affiliations:** 1 Cardiology, Edinburgh Heart Centre, Royal Infirmary of Edinburgh, Little France Crescent, Edinburgh, EH16 4SA, UK; 2 Primary Palliative Care Research Group, Usher Institute for Population Health Sciences and Informatics, University of Edinburgh, Medical School, Teviot Place, Edinburgh, EH8 9AG, UK; 3 Edinburgh Clinical Trials Unit, University of Edinburgh, Outpatients Building, Floor Two, Room D36, Western General Hospital, Crewe Road South, Edinburgh, EH4 2XU, UK; 4 Cardiology Department, Glasgow Royal Infirmary, Castle Street, Glasgow, G4 0SF, UK; 5 Edinburgh Health Services Research Unit, Usher Institute for Population Health Sciences and Informatics, University of Edinburgh, Medical School, Teviot Place, Edinburgh, EH8 9AG, UK

## Abstract

Future Care Planning (FCP) rarely occurs in patients with heart disease until close to death by which time the potential benefits are lost. We assessed the feasibility, acceptability and tested a design of a randomised trial evaluating the impact of FCP in patients and carers. 50 patients hospitalised with acute heart failure or acute coronary syndrome and with predicted 12 month mortality risk of >20% were randomly allocated to FCP or usual care for 12 weeks upon discharge and then crossed-over for the next 12 weeks. Quality of life, symptoms and anxiety/distress were assessed by questionnaire. Hospitalisation and mortality events were documented for 6 months post-discharge. FCP increased implementation and documentation of key decisions linked to end-of-life care. FCP did not increase anxiety/distress (Kessler score -E 16.7 (7.0) vs D 16.8 (7.3), p = 0.94). Quality of life was unchanged (EQ5D: E 0.54(0.29) vs D 0.56(0.24), p = 0.86) while unadjusted hospitalised nights was lower (E 8.6 (15.3) vs D 11.8 (17.1), p = 0.01). Qualitative interviews indicated that FCP was highly valued by patients, carers and family physicians. FCP is feasible in a randomised clinical trial in patients with acute high risk cardiac conditions. A Phase 3 trial is needed urgently.

A holistic palliative care approach is now recommended in international guidelines as a key component of care for patients with advanced chronic heart failure^1^,^2^,^3^. However, numerous reports internationally suggest inadequate delivery of such care^4^,^5^,^6^. Barriers include a lack of randomised trials, a deeply engrained culture of curative care among cardiologists and the challenges of spending longer talking with patients and their families about their future care needs and goals.

Palliative care interventions have been shown to improve outcomes in cancer with one trial suggesting a mortality benefit^7^ and several others showing improvements in symptoms and quality of life (QoL)^8^,^9^,^10^. In heart failure, small studies have demonstrated improvements in breathlessness using opioids^11^ and oxygen^12^. Trials of palliative care for advanced illness, including heart failure among others, have shown mixed results with one showing no impact on symptoms or QoL^13^, and another showing clear improvements in QoL and resource utilisation^14^. Trials focusing on heart failure have also shown improvements in QoL, symptoms and depression^15^,^16^. These trials however have been single centre and have been relatively small. There remains ongoing debate as to when and how palliative care should be introduced to cardiac patients. Further trials are ongoing^17^.

However, focusing exclusively on patients with heart failure excludes a wider group of people with advanced heart disease, including those with coronary disease and valvular heart disease that could also benefit from care planning. The increased use of transcatheter aortic valve implantation for very elderly and often frail people has identified another group whose care planning can be challenging.

In this novel Phase 2 randomised controlled trial we aimed to assess the feasibility and acceptability (to patients and carers) of a Future Care Planning (FCP) intervention in patients with different forms of advanced heart disease. The intervention was a complex intervention, with the trial being designed in line with Medical Research Council guidance^18^ and informed by extensive consultation with patient groups and healthcare professionals using a series of semi-structured interviews^19^. Qualitative aspects of the intervention and the views of patients, carers and general practitioners (GPs) were evaluated using interviews.

## Results

**Figure 1:**
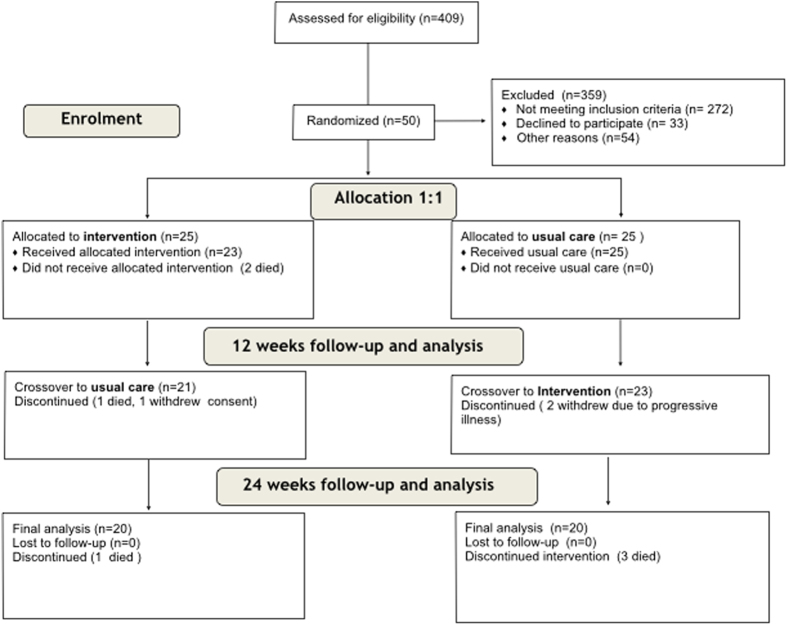
Flow chart of patients screened, excluded and recruited. Patients over the age of 70 were routinely screened on cardiology and general medical wards using the GRACE and EFFECT scores; an estimated risk of death within 12 months of at least 20% was the threshold level for trial inclusion the reasons for failing to recruit eligible patients are also listed in the flow chart.

Over a 12 month period between 1^st^ October 2013 and 31^st^ September 2014, 408 patients were screened, 137 met eligibility criteria of which fifty were randomised to an early or delayed intervention. Reasons for not enrolling eligible patients are outlined in Fig. 1. The characteristics of enrolled patients are summarised in Table 1. For comparison, similar data for the screened population is provided in supplementary Table 1. Study patients were elderly with increased care needs as defined by a high Charleson Comorbidity Index, low mean Karnofsky Performance Scale and more than 50% of the cohort was considered frail according to the Canadian Frailty Scale. There was a low attrition rate for a palliative care study in such a frail population.

Heart failure was the primary diagnosis in 68% (n = 34) of patients; 14% (n = 7) with preserved and 54% (n = 27) with reduced left ventricle ejection fraction. There were no patients with an internal cardiac defibrillator (ICD). The remaining patients had aortic stenosis (n = 5, 10%) and acute coronary syndrome (n = 11, 22%). 64% of patients had a nominated carer who participated in the study, 19 in the early group and 13 in the delayed group. Three patients withdrew from the study during the 24 week follow-up period; 2 due to worsening illness and/or admission to hospital (delayed group) and 1 patient withdrew their consent for personal reasons (early group). There were 7 deaths; 4 in the early group and 3 in the delayed group (Fig. 1).

### Process outcomes linked to the Future Care Plan

**Figure 2:**
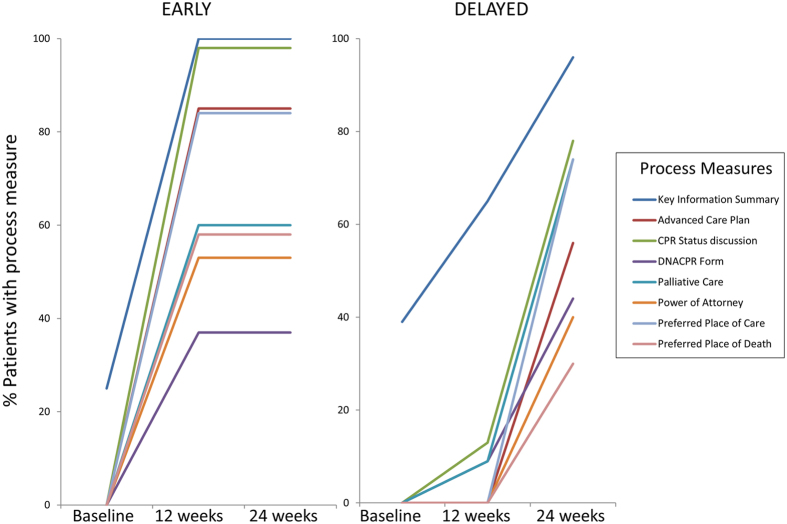
Change in Process Outcomes during the intervention for early and delayed groups. Proportion (%) of patients in early and delayed arms of the trial with completion of each of the process measures included in the intervention which were discussed and documented in the written Future Care Plan at baseline, 12 and 24 weeks after discharge.

Components of the Future Care Plan were achieved more frequently in the early intervention group compared to the delayed intervention group by 12 weeks after discharge (Fig. 2). More patients in the early intervention group had an anticipatory care plan for acute deterioration, a nominated Power of Attorney, a record of CPR discussions, a record of preferred place of care and preferred place of death compared to the delayed group. More patients in the early group were added to the GP palliative care register and more had a care summary shared across primary, secondary and emergency care services by 12 weeks after discharge. These differences had reduced but were still present at 24 weeks following discharge (see supplementary Table 2).

### Patient outcomes - symptoms, QoL and anxiety/distress

The early and delayed treatment groups showed no differences in health related QoL at baseline (EQ5D index (mean(standard deviation)- early: 0.62 (0.27) vs delayed: 0.60 (0.24)) and no significant adjusted mean difference at the 12 or 24 week time points (Table 2).

A similar pattern of results was seen for the EQ5D VAS, symptoms (both composite and individual symptom scores) measured using ESAS and no difference in anxiety/distress assessed using the Kessler Scale.

### Healthcare outcomes

There was no difference in the number of unscheduled readmissions to hospital (Table 3) or unscheduled cardiovascular readmissions at 12 weeks or 6 months after discharge. Time to first readmission was also not significantly different between those who received the intervention in the first 12 weeks after discharge and those who had delayed intervention (hazard ratio 1.05, 95% confidence interval (CI) [0.34, 3.27]; p = 0.93). The duration of hospital stay, expressed as total days spent in hospital was reduced in the early intervention group compared to the delayed group at 12 and 24 weeks after discharge. However, further analysis indicated that the reduced mean hospital stay in the early intervention group was attributable to a slight excess of deaths in that group. By 12 weeks there were 3 deaths in the early group and none in the delayed group. By 24 weeks there was one further death in the early group and 3 in the delayed group (Fig. 1). With regard to place of death, more patients in the early intervention group died in hospital (3 patients) compared to the delayed group (1 patient), while 1 patient in the early group died at home and none in the delayed group (Table 3). These numbers are small, as expected in this phase II trial, and statistical analysis does not indicate a difference. The overall death rate was comparable to that predicted by the prognostic scoring tools used at time of recruitment.

### Carer outcomes

19 carers from the early intervention group and 13 from the delayed intervention group contributed to questionnaire data on QoL, anxiety/distress and caregiver burden at 5 time points during the trial. At the key analysis time point of 12 weeks, where the early group had received the intervention and the delayed group had not, there was no difference in mean QoL score, anxiety/distress score and caregiver burden between the intervention groups (Table 4).

### Qualitative findings

Key findings from interviews with patients, family members and GPs are presented in Appendix 1. Patients appreciated the ongoing contact and communication. Carers expressed a very positive experience mainly arising from the openness generated by the detailed future planning on a number of difficult issues. General practitioners also welcomed the initiation of end of life discussions by the hospital based cardiology team.

## Discussion

This Phase 2, randomised controlled trial was completed successfully and provides valuable data to inform the design of a phase 3 trial. The findings have highlighted a need and have tested a design for an adequately powered trial in this area. In general patients and their carers were supportive, willing and enthusiastic to participate. In the early stages of the study, some patients were excluded by their own cardiologist due to concerns about having open conversations about death and dying. However, there was a progressive change in attitude such that towards the end of the recruitment phase the research team started to receive active referrals from consultant cardiologists. While this change could reflect a number of complex issues, it is likely to reflect improvements in understanding, acceptance and trust between the clinical and research teams as the trial progressed.

An important issue addressed in this feasibility study relates to recruitment and retention of patients. By focusing the screening process on elderly patients (>70 years) with acute cardiac conditions, we were able to identify a large number of eligible patients in a relatively short time period (12 months). Since only one patient chose to actively withdraw from the study, despite discussing many challenging life and death issues, we can conclude that patients and their families found discussions with well trained professionals to be helpful and supportive. The questionnaire data confirmed this as there was no increase in anxiety/distress scores throughout the study among patients or carers. Indeed, it could be the case that the FCP intervention prevented increased distress over time. There was no significant impact of timing of the FCP intervention (early versus delayed) on trial outcomes. There was a slight effect of timing of the intervention on retention of patients, in the delayed group two withdrew due to progressive illness before the FCP intervention was delivered. These two patients effectively switched to “open- label” palliative care. This is not an unexpected finding in a randomised controlled trial of care planning in advanced heart disease and its impact in a larger trial will need to be carefully considered with respect to design and sample size.

This study has also supported the methodology of recruiting patients at the time of hospital discharge following an unscheduled hospital admission. This is a key opportunity to begin discussions with patients and their families about issues that might otherwise be difficult to address, including cardiopulmonary resuscitation and its benefits and harms.

We included a broad range of people with cardiac conditions (acute coronary syndrome, valvular heart disease and all forms of heart failure) in the trial, many with co-morbidities. This is both equitable and important as we clearly identified a population at high risk of readmission and death over the 6 months following index admission. The high mortality risk applies to heart failure with reduced ejection fraction, normal ejection fraction and in people with predominantly valvular heart disease. High mortality risk is also present in patients with acute coronary syndrome particularly accompanied by transient or persistent features of heart failure. These patients are well recognised to be at high risk of future clinical events^20^,^21^. However, unlike the HF patients most of the ACS patients were free of long term symptoms prior to admission. While this could have affected patient perspective with regard to Future Care Planning, qualitative interviews suggested that both ACS and HF patients were equally receptive to discussions regarding end of life issues.

Creation of a written Future Care Plan, agreed with the patient and their family, and shared electronically with the patient and other healthcare organisations was also a key component of the trial. Used in this way a FCP can support person-centred decision making about treatment and care options in community and hospital settings and represents a robust shared decision-making process involving patients and carers. Discussing and formulating the FCP took time and this can be difficult to manage in average clinical circumstances. The series of three separate 1 hour-long meetings spanning a 12 week period that continued while the patient was at home were important for this process. The challenges of adopting FCP processes in routine clinical practice need to be pragmatic and carefully considered as we move forward to a larger trial.

Use of generic questionnaires such the ESAS, EQ5D, EQ5D-VAS, Kessler was not burdensome for patients and the response rates were surprisingly high. However, the data generated in this small cohort was not sensitive or specific enough to observe a positive effect of the intervention. These issues and the relatively high mortality during follow-up limit the utility of this type of data as a trial endpoint. The use of heart failure specific QoL questionnaires may have detected more subtle changes^22^ or possibly a questionnaire designed to detect changes in symptoms and concerns towards the end of life^23^.

Re-admission to hospital and bed-utilisation represent important outcomes which merit consideration for a future trial. Hospitalisation is well known to impact on QoL and any reduction generated by a holistic care intervention should, by inference, lead to improved QoL. Although the mean number of nights spent in hospital was lower in the early intervention group further analysis and correction for the small difference in mortality between groups showed that the number of days spent alive and out-of-hospital was not different between the two arms of the study.

A number of previous studies have used a holistic care intervention in patients with heart failure^14^,^15^,^16^. However, this study differs in several important ways. Firstly, we targeted patients with a range of heart conditions, including heart failure. Secondly, we used a risk-threshold based on prognostic scoring tools to select patients for the study. These scoring tools are recognised to be highly predictive in cohorts of patients but less predictive for individual patients^24^. However, our aim was not to make an accurate prognostic judgment about life expectancy for an individual patient but rather to identify a population of patients at high risk of death or deterioration in their health and who may therefore benefit from additional holistic care. Our trial has shown quite clearly that these prognostic tools can provide basis for selecting high risk patients with a high burden of comorbidity as illustrated by the high Charlson, frailty and Karnofsky scores observed in those recruited.

Finally, we created and shared a Future Care Plan between hospital, GP and the emergency care services using an electronic record called the Key Information Summary (KIS). This is a fundamental component of any intervention which aims to alter the experience of the patient and their use of healthcare services in the final phase of an advanced illness. It is an approach which has been much called for by a number of researchers in this field^25^,^26^.

This novel phase 2 trial has confirmed that a randomised trial approach using FCP is feasible and acceptable to patients with a range of heart disease conditions associated with high risk of death. The intervention clearly improved the documentation of a number of key issues linked to patient-centred healthcare. It has defined components necessary for a larger, adequately powered clinical trial with the potential to influence end of life care for people with advanced heart disease.

## Methods

The study protocol was approved by the Lothian Research Ethics Committee (reference 12/SS/0223) and conducted in collaboration with a UKCRC-registered Clinical Trials Unit. Patients were recruited, consented and randomised by the trial nurse according to the approved protocol and in accordance with Good Clinical Practice guidelines. Recruitment was undertaken in cardiology, medical and care of the elderly hospital wards following an unscheduled hospital admission with heart failure and or acute coronary syndrome based on European Society of Cardiology guidelines^1^,^27^. Eligibility criteria included a predicted 12 month mortality risk of 20% or greater estimated using the Global Registry of Acute Coronary Syndrome (GRACE) score for ACS^28^ and the Enhanced Feedback for Effective Cardiac Treatment (EFFECT) score for heart failure and patients with aortic stenosis who presented with heart failure^29^. Exclusion criteria included moderate/severe dementia, prognosis less than 30 days and those already on a palliative care register. Enrolled patients were asked to identify an informal carer to take part in the study. Patients and carers gave written informed consent and knew they could withdraw from the study at any time. Patients were randomised using a crossover design in a 1:1 ratio using random permuted blocks to receive one of two care pathways: pathway 1 included 12 weeks of a Future Care Plan intervention (FCP) at discharge followed by crossover to 12 weeks of usual care (early group) or pathway 2 which included 12 weeks of usual care followed by cross over to 12 weeks of FCP (delayed group). This design was ethical in that it ensured that all patients received the FCP intervention and also allowed us to assess potential impact of the timing of the intervention on process and outcomes.

Questionnaires were completed at 5 time points by patients and carers - baseline (at discharge), 6, 12, 18 and 24 weeks after discharge in the home setting. Response rates were high with 95% of surviving patients and 80% of carers providing responses at each of the 5 time points.

### FCP Intervention

The intervention lasted 12 weeks and had three main components:

1. An initial, one hour semi-structured meeting with the trial cardiologist (MD) and the trial nurse specialists involving the patient and their carer; followed by two 1 hour meetings with the trial nurse in the patient’s home at 6 and 12 weeks.
2. Discussion and documentation of an agreed personal Future Care Plan which was sent to each patient and uploaded by the general practitioner using the electronic KIS (used in Scotland to routinely communicate with out- of-hours and hospital services http://www.snughealth.org.uk/gp-software/key-information-summary).
3. Ongoing telephone support (available Monday to Friday, 9am–5pm) from the trial nurse for the 12 weeks offering advice, support and information about their healthcare and social needs.

In preparation for the study, the cardiologist and the trial nurse specialist attended an advanced communication workshop that included experiential training FCP. They also had short clinical attachments with community palliative care teams involving FCP.

The first interview/meeting focused on the information needs, key concerns and priorities of the patient and carer using a semi-structured interview guide to provide a consistent framework. The key topics discussed were: exploring their experiences of being in hospital, what treatments had been given or changed, their understanding and expectations about their condition, nominating a Power of Attorney or a surrogate decision maker, preferences for place of care should they deteriorate in the future (including place of care when dying) and likely outcomes of treatment; specifically discontinuing life-prolonging treatments and cardiopulmonary resuscitation. The final FCP document also included a detailed anticipatory care plan describing actions to be taken if the patient’s health deteriorated acutely. A paper copy of the FCP was posted to the patient after this first meeting and sent to the GP. The trial team also recommended to the GP that the patient was added to the practice palliative care register. At the 6 week meeting, the patient’s condition and their views and perceptions about their health and care needs were explored once again by the trial nurse. Any concerns or needs not raised during the first meeting were discussed. The patient’s FCP was reviewed with them and their carer and any changes agreed and noted. The updated version of the FCP was again sent back to the patient and to their GP practice for upload into the KIS. The 12 week meeting focused on plans for handing care coordination over to the primary care team and ongoing use of the FCP as part of this. In all cases, the trial nurse phoned the GP practice to try and speak with the patient’s GP to ensure that information about the patient’s current and Future Care Plans were communicated effectively. A final version of the FCP was sent to the GP for upload to the KIS. Completed Do Not Attempt Cardiopulmonary Resuscitation (DNACPR) forms were held by the patient and a special note added to the KIS.

### Outcome measures

Patients completed QoL questionnaires (EQ5D)^30^ before discharge and at 6, 12, 18 and 24 weeks. EQ-5D dimension scores were converted to index scores using UK population values^31^. They also completed a symptom scoring tool (Edmonton Symptom Assessment Scale (ESAS)^32^ and a stress/anxiety questionnaire (Kessler)^33^ at these same time points. Carers were asked to complete the EQ-5D, EQ5D-Visual analogue scale (Health-thermometer), Kessler Distress Score and the ZARIT6^34^, assessing caregiver burden, at each time point. All scheduled and unscheduled hospital admissions and all-cause and disease specific mortality were obtained from electronic health records over the 6 month period following recruitment. The primary outcome variable was health related QoL (EQ5D index) measured at the 12 week time point.

A range of other clinical and demographic data was collected from the patient’s care record including data required to complete the Charleson Comorbidity Index^35^, the Canadian Study of Health and Ageing Clinical Frailty Scale^36^ and the Karnofsky Performance Scale^37^.

Qualitative interviews were undertaken as part of the post-trial evaluation to assess the views of patients (n = 12), carers (n = 8) and general practitioners (GPs, n = 6) about the trial. Patients were selected randomly with a similar number in the early and delayed groups. All interviews were digitally recorded, transcribed verbatim and analysed thematically with the assistance of computer software (Nvivo) designed for this purpose^38^.

### Statistical analysis

The study sample size was chosen as a pragmatic number that would allow us to explore recruitment and retention of patients, a range of outcomes and the feasibility of a randomised trial approach. The primary outcome was mean quality of life (EQ5D-index) at 12 weeks comparing patients receiving early and delayed intervention. This was assessed using analysis of covariance (ANCOVA) adjusting for the baseline EQ5D-index. The intervention effect is presented as the adjusted mean difference (for early minus delayed intervention), its 95% confidence interval (CI) and the p-value associated with the test of the null hypothesis that the adjusted mean difference equals zero. Secondary patient-outcomes included EQ5D-index at 24 weeks, EQ5D-VAS, symptom score and anxiety/distress at 12 and 24 weeks. Carer-outcomes formed part of an exploratory analysis. Secondary patient-outcomes and carer outcomes were analysed for continuous outcomes using the same method as the primary outcome using ANCOVA and adjusting for the baseline measurement. Time to event patient-outcomes were analysed using the Cox proportional hazards model and reported using a hazard ratio and 95% CI. Outcomes representing count data were analysed by Poisson regression and reported using risk ratio and 95% CI. Outcomes representing binary data were analysed by logistic regression and reported as odds ratio and 95% CI.

## Additional Information

**How to cite this article**: Denvir, M. A. *et al*. Phase 2 Randomised Controlled Trial and Feasibility Study of Future Care Planning in Patients with Advanced Heart Disease. *Sci. Rep.* **6**, 24619; doi: 10.1038/srep24619 (2016).

## Supplementary Material

Supplementary Information

## Figures and Tables

**Table 1:** Baseline Patient Characteristics.

	**Early (n = 25)**	**Delayed (n = 25)**
Age in years, Mean (SD)	81.9 (7.1)	80.2 (10.0)
Gender, Male (n, %)	17 (68.0)	13 (52.0)
Smoking Status (n, %)		
Never	15 (60.0)	10 (40.0)
Previous	8 (32.0)	14 (56.0)
Current	1 (4.0)	0 (0.0)
Formal Carer (n, %)	4 (16.0)	4 (16.0)
Informal Carer in Trial (n, %)		
None	6 (24.0)	12 (48.0)
Daughter/son	6 (24.0)	6 (24.0)
Partner/Spouse	11 (44.0)	6 (24.0)
Other Family	2 (8.0)	1 (4.0)
Primary Diagnosis (n, %)		
ACS	8 (32.0)	3 (12.0)
Heart Failure	14 (56.0)	20 (80.0)
Valvular Disease	3 (12.0)	2 (8.0)
Comorbidities (n, %)		
CPD	6 (24.0)	8 (32.0)
CVD	5 (20.0)	2 (8.0)
Cancer	5 (20.0)	4 (16.0)
CKD	13 (52.0)	18 (72.0)
Dementia	0 (0.0)	1 (4.0)
Diabetes	9 (36.0)	10 (40.0)
Liver Disease	0 (0.0)	1 (4.0)
Routine Bloods, (Mean (SD))		
Creatinine (umol/l)	134.5 (65.0)	186.9 (120.4)
Hemoglobin (g/l)	119.9 (20.7)	115.4 (19.2)
Sodium (mmol/l)	136.5 (5.0)	137.0 (6.7)
Urea (mmol/l)	12.3 (7.0)	17.1 (8.6)
eGFR (mls/min)	50.1 (22.6)	34.5 (18.6)
Moderate or Severe LVSD (n, %)	16 (64.0)	14 (56.0)
Co-morbidity score		
Karnofsky Score (Mean (SD))	62.8 (13.1)	61.6 (11.8)
Charlson Comorbidity Index (Mean (SD))	3.9 (1.8)	4.5 (1.5)
Frail (%)	13 (52.0)	15 (60.0)

Abbreviations: SD, standard deviation; N, number of observations; CKD, chronic kidney disease; CPD, chronic pulmonary disease; CVD, cerebrovascular disease; ACS, acute coronary syndrome; EF, ejection fraction; eGFR, estimated glomerular filtration rate; LVSD, left ventricular systolic dysfunction.

**Table 2:** Patient Questionnaire Outcomes.

	**Early (n = 25)**		**Delayed (n = 25)**		**Adjusted Mean**		
	**N**	**Mean (SD)**	**N**	**Mean (SD)**	**Difference (95%CI)**	**P-Value**	
12 Weeks							
EQ5D Index	21	0.54 (0.29)	23	0.56 (0.24)	−0.01 (−0.16,0.13)	0.86	
EQ5D VAS	19	60.9 (21.2)	22	57.0 (19.7)	4.75 (−5.86,15.37)	0.37	
Edmonton Symptom Scale	19	24.5 (12.5)	23	23.5 (14.7)	0.62 (−8.34,9.58)	0.89	
Kessler Distress Scale	17	16.7 (7.0)	22	16.8 (7.3)	0.17 (−4.13,4.46)	0.94	
24 Weeks							
EQ5D Index	20	0.45 (0.31)	20	0.55 (0.23)	−0.07 (−0.25,0.11)	0.44	
EQ5D VAS	17	64.6 (19.1)	20	67.3 (16.3)	−0.42 (−12.7,11.89)	0.95	
Edmonton Symptom Scale	17	21.8 (16.1)	20	18.8 (12.5)	3.18 (−6.90,13.26)	0.52	
Kessler Distress Scale	17	16.5 (6.2)	17	13.6 (2.0)	2.70 (−0.73,6.12)	0.12	

Note. Mean difference for early minus delayed intervention adjusted for baseline values. Abbreviations: N, number of observation; SD, standard deviation; CI, confidence interval; VAS, visual analogue scale.

**Table 3:** Healthcare outcomes.

	**Early (n = 25)**	**Delayed (n = 25)**	**Risk Ratio**	**P**
	**Mean (SD)**	**Mean (SD)**	**(95% CI)**	
12 Weeks				
All Unscheduled Admissions	0.5 (0.9)	0.4 (0.6)	1.25 (0.54,2.89)	0.6
Cardiovascular Unscheduled Admissions	0.3 (0.7)	0.2 (0.5)	1.22 (0.41,3.62)	0.73
Nights in Hospital	2.7 (5.5)	5.4 (9.4)	0.50 (0.37,0.67)	<0.01
6 Months				
All Unscheduled Admissions	0.8 (1.3)	0.7 (0.7)	1.23 (0.64,2.34)	0.54
Cardiovascular Unscheduled Admissions	0.3 (0.8)	0.4 (0.6)	0.83 (0.33,2.11)	0.7
Nights in Hospital	8.6 (15.3)	11.8 (17.1)	0.73 (0.61,0.88)	<0.01
Mortality (n(%))			Hazard Ratio (95% CI)	
12 weeks	3 (12.0)	0 (0.0)	N/A	N/A
24 weeks	4 (16.0)	3 (12.0)	1.41 (0.32,6.30)	0.65
First Hospital Re-Admission (n(%))				
Within 12 weeks	6 (24.0)	7 (28.0)	1.05 (0.34,3.27)	0.93
Within 6 months	13 (52.0)	14 (56.0)	0.94 (0.44,2.04)	0.89
Actual Place of Death (n(%))				
Hospital	3 (75)	1 (33.3)	–	–
Hospice	0 (0.0)	1 (33.3)	–	–
Care Home	0 (0.0)	1 (33.3)	–	–
Home	1 (25)	0 (0.0)	–	–

Note. Hazard Ratio not applicable (N/A) due to zero events in the delayed intervention groups at 12 weeks. Place of death was analysed using descriptive statistics only. Abbreviations: N, number; SD, standard deviation; CI, confidence interval.

**Table 4:** Carer Questionnaire Outcomes.

	**Early (n = 19)**		**Delayed (n = 13)**		**Adjusted Mean Difference**	
	**N**	**Mean (SD)**	**N**	**Mean (SD)**	**(95% CI)**	**P**
12 Weeks						
EQ5D Index	14	0.82 (0.13)	9	0.80 (0.20)	−0.05 (−0.16,0.06)	0.38
EQ5D VAS	15	83.5 (12.3)	10	81.8 (15.9)	−3.08 (−11.7,5.50)	0.46
Carer-giver Burden (Zarit 6)	15	5.9 (6.2)	9	5.2 (6.3)	0.84 (−2.53,4.21)	0.61
Kessler Distress Scale	15	14.3 (3.9)	10	15.8 (7.7)	−0.79 (−3.55,1.97)	0.56
24 Weeks						
EQ5D Index	12	0.80 (0.13)	10	0.82 (0.18)	−0.06 (−0.20,0.07)	0.32
EQ5D VAS	12	79.6 (14.9)	10	83.1 (17.1)	−5.81 (−15.7,4.03)	0.23
Carer-giver Burden (Zarit 6)	11	6.5 (6.4)	10	4.8 (6.3)	1.70 (−1.43,4.83)	0.27
Kessler Distress Scale	11	15.3 (3.5)	10	16.1 (10.8)	−1.68 (−4.47,1.11)	0.22

Note. Mean difference for early minus delayed intervention adjusted for baseline values. Abbreviations: N, number of observations; SD, standard deviation; VAS, visual analogue scale.
