# Supplementary material for: Phase 2 Randomised Controlled Trial and Feasibility Study of Future Care Planning in Patients with Advanced Heart Disease
Source: Sci Rep. 2016 Apr 19;6:24619. doi: 10.1038/srep24619 (PMC4836296; doi:10.1038/srep24619)
Supplement: Supplementary Information [file srep24619-s1.pdf]

Phase 2 Randomised Controlled Trial and Feasibility Study of Future Care Planning in Patients with Advanced Heart Disease

Martin A Denvir, Sarah Cudmore, Gill Highet, Shirley Robertson, Lisa Donald, Jacqueline Stephen, Kristin Haga, Karen Hogg, Christopher J Weir, Scott A Murray, Kirsty Boyd

Supplementary Table 1: Patient Characteristics of Screened versus Enrolled Patients

	Screened (n=359)	Enrolled (n=50)
Age in Years, Mean (SD)	78.7 (7.2)	81.1 (8.6)
Gender, Male (%)	212 (59.1)	30 (60.0)
**Smoking Status (%)**		
Never	120 (33.4)	25 (50.0)
Previous	173 (48.2)	22 (44.0)
Current	42 (11.7)	1 (2.0)
**Primary Diagnosis (%)**		
ACS	233 (64.9)	11 (22.0)
Heart Failure (all)	126 (35.1)	34 (68.0)
Valvular Disease	0 (0.0)	5 (10.0)
**Comorbidities (%)**		
Anaemia	13 (3.6)	9 (18.0)
COPD	20 (5.6)	10 (20.0)
Cancer	20 (5.6)	11 (22.0)
CVA	19 (5.3)	6 (12.0)
CKD	104 (29.0)	31 (62.0)
Cirrhosis	2 (0.6)	2 (4.0)
Mild Dementia	3 (0.8)	2 (4.0)
**Unscheduled Admissions, Mean (SD)**	0.7 (1.3)	1.4 (2.4)
**Routine Bloods, Mean (SD)**		
Creatinine (umol/l)	107.5 (46.5)	160.7 (99.3)
Hemoglobin (g/l)	128.9 (19.8)	117.6 (19.9)
Sodium (mmol/l)	137.6 (4.3)	136.7 (5.9)
Urea (mmol/l)	11.6 (47.6)	14.7 (8.1)
eGFR (mls/min)	62.1 (24.0)	42.3 (21.9)
**Moderate or Severe LVSD (%)**	100 (28.0)	30 (60.0)
**Estimated Mortality Risk, Mean (SD)**		

GRACE %	17.2 (12.9)	52.0 (20.7)
EFFECT	105.5 (30.5)	130.5 (44.0)
**Co-morbidity score**		
Karnofsky Score, Mean (SD)	74.1 (19.0)	62.2 (12.3)
Charlson Comorbidity Index, Mean (SD)	3.1 (1.9)	4.2 (1.7)
Frail (%)	100 (27.9)	28 (56.0)

Abbreviations: SD, standard deviation; N, number of observations; CKD, chronic kidney disease; COPD, chronic obstructive pulmonary disease; CVA, cerebrovascular accident; ACS, acute coronary syndrome; EFFECT, Enhanced Feedback for Effective Cardiac Treatment; GRACE, Global Register of Acute Coronary Events; EF, ejection fraction; eGFR, estimated glomerular filtration rate; LVSD, left ventricular systolic dysfunction.

Supplementary table 2: Process outcomes data at 12 and 24 weeks after discharge (see figure 2)

	Early (n=19)N (%)	Delayed (n=23) N (%)	Difference in Proportion (95% CI)	Odds Ratio (95% CI)	P-value
**12 Weeks**					
Key Information Summary Shared	19 (100.0)	15 (65.2)	34.8 (15.3,54.2)	N/A	-
Advanced Care Plan Shared	16 (84.2)	0 (0.0)	84.2 (67.8,100.0)	N/A	-
CPR Status Shared	19 (100.0)	3 (13.0)	87.0 (73.2,100.0)	N/A	-
DNACPR Form Shared	7 (36.8)	2 (8.7)	28.1 (3.6,52.7)	6.1 (1.1,34.3)	0.02
Palliative Care Status Shared	11 (57.9)	2 (8.7)	49.2 (24.2,74.2)	14.4 (2.6,80.0)	<.01
Power of Attorney Shared	10 (52.6)	0 (0.0)	52.6 (30.2,75.1)	N/A	-
Preferred Place of Care Shared	16 (84.2)	0 (0.0)	84.2 (67.8,100.0)	N/A	-
Preferred Place of Death Shared	11 (57.9)	0 (0.0)	57.9 (35.7,80.1)	N/A	-
**24 Weeks**					
Key Information Summary Shared	19 (100.0)	22 (95.7)	4.3 (-4.0,12.7)	N/A	-
Advanced Care Plan Shared	16 (84.2)	13 (56.5)	27.7 (1.6,53.8)	4.1 (0.9,18.1)	0.05
CPR Status Shared	19 (100.0)	18 (78.3)	21.7 (4.9,38.6)	N/A	-
DNACPR Form Shared	7 (36.8)	10 (43.5)	-6.6 (-36.3,23.0)	0.8 (0.2,2.6)	0.66
Palliative Care Status Shared	11 (57.9)	17 (73.9)	-16.0 (-44.6,12.5)	0.5 (0.1,1.8)	0.27
Power of Attorney Shared	10 (52.6)	9 (39.1)	13.5 (-16.5,43.5)	1.7 (0.5,5.9)	0.38
Preferred Place of Care Shared	16 (84.2)	17 (73.9)	10.3 (-14.0,34.6)	1.9 (0.4,8.8)	0.41
Preferred Place of Death Shared	11 (57.9)	7 (30.4)	27.5 (-1.6,56.6)	3.1 (0.9,11.2)	0.07

Note. Outcomes refer to being shared on the Key Information Summary (KIS). Denominator for the early intervention group is 19 and 23 for the delayed intervention group due to no access to the KIS for some patients through withdrawn consent or death. Difference in proportion for early minus delayed intervention. Odds ratio for early:delayed intervention. Odds ratios are not applicable (N/A) if 0 or 100% were observed in the intervention groups.
Abbreviations: CI, confidence interval; (DNA)CPR- do not attempt cardiopulmonary resuscitation.
